# Insulin-like growth factors and risk of kidney cancer in men

**DOI:** 10.1038/sj.bjc.6605722

**Published:** 2010-06-01

**Authors:** J M Major, M N Pollak, K Snyder, J Virtamo, D Albanes

**Affiliations:** 1 Division of Cancer Epidemiology and Genetics, National Cancer Institute, NIH, Bethesda, Maryland 20852, USA; 2 Department of Oncology, Jewish General Hospital and McGill University, Montreal, Quebec, Canada H3T 1E2; 3 Information Management Services Inc., Silver Spring, Maryland 20904, USA; 4 Department of Chronic Disease Prevention, National Institute for Health and Welfare, Helsinki FI-00300, Finland

**Keywords:** insulin-like growth factor, IGF-I, incidence, men

## Abstract

**Background:**

Insulin-like growth factor-I (IGF-I) has been shown to increase kidney growth, glomerular filtration rate, and renal function.

**Methods:**

In the prospective Alpha-Tocopherol, Beta-Carotene Cancer Prevention (ATBC) study of 29 133 Finnish male smokers aged 50–69 years, serum concentrations of IGF were measured in samples collected in 1985–1988. A total of 100 men with kidney cancer diagnosed ⩾5 years after blood collection through 1997 were compared with a subcohort of 400 men; logistic regression models were used to estimate the risk of developing kidney cancer.

**Results:**

Men with IGF-I levels >113 ng ml^−1^ were 59% less likely to develop kidney cancer than men with levels ⩽113 ng ml^−1^ (odds ratio=0.41; 95% confidence interval=0.23–0.75). The IGF binding protein-3 (IGFBP-3) levels did not alter the association. No association was observed between IGFBP-3, or molar ratio of IGF-I/IGFBP-3, and kidney cancer.

**Conclusions:**

Low serum IGF-I levels in this cohort of older middle-aged male smokers are associated with increased kidney cancer risk, independent of IGFBP-3.

Kidney cancer, comprised of cases including both clear cell renal cell carcinoma (RCC) and transitional cell carcinoma of the renal pelvis, is the seventh and ninth most common cancer in men and women, respectively, in the United States, with an estimated 58 000 diagnoses and 13 000 deaths in 2009 (American Cancer Society, 2009). Incidence is highest in those ages 50–70 years, and almost twice as high in men as in women (Parkin *et al*, 2002). Two established risk factors include smoking, with smokers being twice as likely as nonsmokers to develop RCC and four times as likely to develop cancer of the renal pelvis, and obesity (Chow *et al*, 2000; Murai and Oya, 2004; McLaughlin *et al*, 2006; Pischon *et al*, 2006; World Cancer Research Fund/American Institute for Cancer Research, 2007). Increased bioavailable insulin-like growth factor-I (IGF-I) levels may be one mechanism through which obesity increases kidney cancer risk.

Insulin-like growth factor-I is a single-chain polypeptide, secreted primarily by the liver in response to growth hormone that serves important functions in normal growth, development, and metabolism. It acts through endocrine, paracrine, and autocrine mechanisms (LeRoith *et al*, 1992; Jones and Clemmons, 1995) to influence cell proliferation, differentiation, and apoptosis in a variety of tissues (Pollak, 2000; Moschos and Mantzoros, 2002; Juul, 2003). Higher serum levels have been associated with increased risk of cancers of the prostate, colon, breast, and lung (Chan *et al*, 1998; Hankinson *et al*, 1998; Ma *et al*, 1999; Yu *et al*, 1999; Wakai *et al*, 2002; Renehan *et al*, 2004; Rowlands *et al*, 2009). The impact of IGF-I is influenced by IGF binding protein-3 (IGFBP-3), the most abundant high-affinity BP in circulation that binds >75% of all IGF-I (Ferry *et al*, 1999; Cheung *et al*, 2004). In the kidney, IGF-I has been shown to increase growth of the microvasculature and the glomerular filtration rate, and administration of IGF-I has been shown to increase renal function and been proposed as a possible therapeutic agent in chronic renal failure (Hirschberg and Adler, 1998). We conducted a case–cohort study nested within the Alpha-Tocopherol, Beta-Carotene Cancer Prevention (ATBC) study of Finnish male smokers to examine the prospective relation of IGF-I and IGFBP-3 to risk of kidney cancer.

## Materials and methods

The ATBC study is a randomized intervention trial that tested whether supplementation with *α*-tocopherol, *β*-carotene (or both) reduced the incidence of lung and other cancers. Details of the ATBC study have been described (The ATBC Cancer Prevention Study Group, 1994). Briefly, the study cohort consisted of 29 133 eligible men residing in southwestern Finland, aged 50–69 years, who smoked at least five cigarettes per day at study entry, with participants being enrolled between 1985 and 1988. Men with a history of cancer were not eligible to participate. The institutional review boards of the National Public Health Institute in Finland and the US National Cancer Institute approved the study protocol, and written informed consent was obtained from the participants before study enrolment.

At study entry, investigators administered questionnaires that collected information on lifestyle behaviours and medical history. Height and weight were measured using standard methods, and body mass index (kg m^−2^) was calculated. Fasting blood samples were collected at the baseline visit and stored frozen at −70°C until analysed. Following separation of IGFs from IGFBPs, total IGF-I and IGFBP-3 were measured in serum by enzyme-linked immunosorbent assays (Diagnostic Systems Laboratory Inc, Houston, TX, USA). The intra-assay coefficient of variation (CV) was 5.2% for IGF-I and 4.2% for IGFBP-3; the inter-assay CV was 4.6 and 6.2%, respectively.

Diagnosis of kidney cancer was defined by the International Classification of Diseases, 9th Revision (ICD-9 codes 189.0, 189.1, and 189.2). Incidence of cancer through the end of 1997 was ascertained through linkage with the Finnish Cancer Registry and study-based reviews; medical records were reviewed by study physicians to confirm cancer diagnosis. During up to 13 years of follow-up, 100 incident cases of kidney cancer were randomly selected from 121 cases diagnosed at least 5 years after the baseline blood collection (i.e., in order to both conserve limited serum resources and reduce reverse causality). To serve as a subcohort comparison group, we randomly selected 400 men from the full ATBC study cohort who were in active study follow-up for at least 5 years and also cancer free at that time. Hence, all the participants in this study were alive and without evidence of cancer during the first 5 years of follow-up.

### Statistical analysis

The IGF-I to IGFBP-3 molar ratio was calculated as a measure of bioavailability using the following conversion: 1 ng ml^−1^ IGF-I =0.130 nmol l^−1^ and 1 ng ml^−1^ IGFBP-3 =0.036 nmol l^−1^. Quartiles of IGF concentrations were calculated based on the distribution in the comparison group.

Descriptive statistics were calculated for baseline characteristics of participants by kidney cancer status; differences between cases and the comparison group were determined using Studentized *t*-tests, Wilcoxon rank sum, and χ^2^-statistics for group comparisons of means, medians, and proportions, respectively. General linear models were used to evaluate the association of potential confounders with quartiles of IGF concentrations among the comparison group. Unconditional logistic regression models were used to estimate the association between quintiles of IGF concentrations and risk of kidney cancer. In addition to evaluating cross-product terms along with the main effects in the models, associations were examined stratified by number of cigarettes smoked per day (⩽20 *vs* >20) and history of hypertension. Trend tests were calculated by including the median of each quartile of serum IGF concentrations as a continuous variable in addition to the covariates in the final multivariable models. Statistical significance was based on two-sided *P*-values of <0.05. All statistical analyses were carried out using SAS version 9.1 (SAS Institute Inc., Cary, NC, USA).

## Results

Baseline characteristics of the study participants according to kidney cancer status are presented in Table 1. Men who developed kidney cancer were slightly older (mean age, 58 *vs* 56 years; *P*=0.03), had lower consumption of alcohol, and smoked more cigarettes daily. In addition, a history of hypertension was significantly more common among men with kidney cancer (28.0 *vs* 18.5%, *P*=0.04), known to be more common in those who are older, overweight, or heavier smokers. Most characteristics did not differ considerably across quartiles of IGF-I and IGFBP-3 (Table 2), although men with higher IGFs were slightly younger, taller, and heavier. Also, higher IGFBP-3 was more common in younger men and those with fewer years of smoking, the latter possibly being explained by its correlation with age. The trial supplementation group assignment was not associated with IGF measures (*P*>0.30, each) or kidney cancer (*P*=0.85) (data not shown).

**Figure 1:**
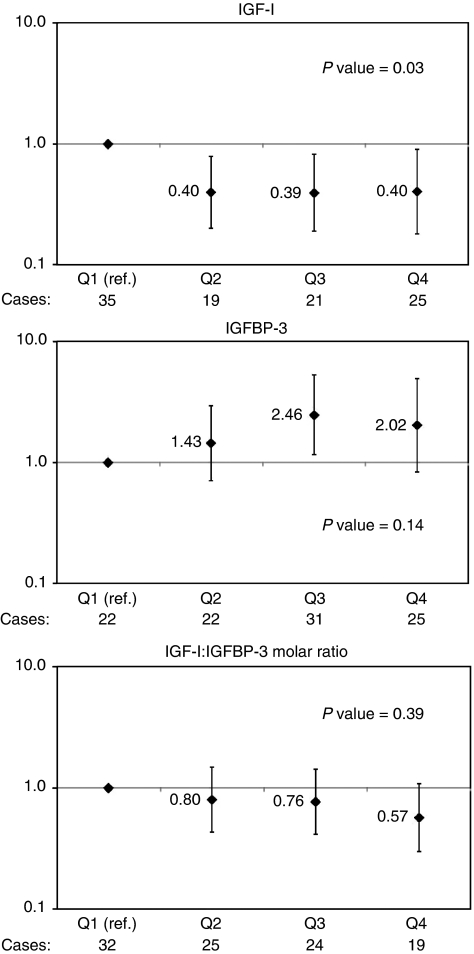
Multivariable-adjusted odds ratios for quartiles of IGF-I, IGFBP-3, and IGF-I/IGFBP-3 molar ratio. Adjusted for age, weight, height, cigarettes per day, and hypertension. IGF-I and IGFBP-3 were simultaneously adjusted for in their respective models. *P*-values for overall effect.

As shown in Figure 1, the multivariable-adjusted odds ratios (ORs) and 95% confidence interval (CIs) for kidney cancer for increasing quartiles of IGF-I were 0.40 (0.20–0.79), 0.39 (0.19–0.81), and 0.40 (0.18–0.90) compared with the low-quartile reference category. The overall association between IGF-I and kidney cancer was statistically significant (Type III Analysis of Effects, *P*=0.03), however, there was no evidence of a dose-risk trend. With regard to the IGFBP-3, the ORs suggest a decreased risk for men in the lowest quartile and a modest trend; however, only men in the third quartile were at significantly increased risk compared with men in the lowest quartile (OR=2.46; 95% CI=1.15–5.27), and the trend test was not significant. For IGF-I/IGFBP-3 molar ratio, overall the association between molar ratio and kidney cancer was not statistically significant (Type III Analysis of Effects, *P*=0.39), nor were any of the individual quartiles when compared with the reference category (Figure 1).

As the ORs for individual IGF-I quartiles 2 through 4 did not materially differ from one another and were in the same direction, we re-examined the associations for quartiles 2–4 combined. An inverse association between IGF-I serum levels and kidney cancer risk was statistically significant (Type III Analysis of Effects, *P*<0.01) when comparing the top three quartiles combined to the lowest quartile. Men with IGF-I levels >113 ng ml^−1^ were 59% less likely to develop kidney cancer than men with IGF-I levels ⩽113 ng ml^−1^ (OR=0.41; 95% CI=0.23–0.75). Exclusion of renal pelvic cancers (*n*=11) yielded similar results (data not shown). No significant interactions were observed between the IGF measures and number of cigarettes smoked daily (⩽20 *vs* >20) (*P*>0.60, both IGF-I and IGFBP-3) or history of hypertension (*P*>0.25, both IGF-I and IGFBP-3).

## Discussion

In this cohort of Finnish male smokers, we found a significant inverse association between serum IGF-I concentrations and risk of kidney cancer and a nonstatistically significant positive association for IGFBP-3. Men with IGF-I levels >113 ng ml^−1^ were 59% less likely to develop kidney cancer than men with lower levels. To our knowledge, this study is the first population-based, epidemiologic investigation to examine the association between serum IGF and kidney cancer.

Our findings are not in accord with experimental studies, in which IGF has been shown to have promoting effects on renal carcinogenesis. *In vitro* studies (Cheung *et al*, 2004) demonstrated that IGF-I stimulated growth of cultured human metastatic RCC cells; however, only weak effects were observed for IGF-I and IGFBP-3 in the nonmetastatic RCC cells. Furthermore, an immunohistochemistry analysis of tissue from 180 RCC patients showed that IGF-I expression was strong in clear cell, but not papillary, tumours, indicating differential expression across histologies (Schips *et al*, 2004). Mixed findings have been reported for IGF-IR, with one study demonstrating increased expression and another failing to detect any expression in human RCC tissue (Ramp *et al*, 1997).

In a clinical study of 256 consecutive RCC patients investigating the relation between serum IGF-I and disease progression and survival (Rasmuson *et al*, 2004), researchers found that male and female patients with serum IGF levels greater than the median value were 38% less likely to die of RCC compared with patients with serum levels below the median (hazard ratio=0.62, 95% CI=0.41–0.95) and that IGF-I levels were inversely proportional to tumour stage and grade. Unfortunately, the small number of cases and the nonroutine ascertainment of tumour characteristics preclude our ability to examine these associations in the ATBC study. The increased risk of kidney cancer associated with lower IGF-I levels we observed in ATBC is consistent with two recent investigations from the ATBC study that found an inverse association of IGF-I levels and incidence of glioma (Lonn *et al*, 2007) and liver cancer (unpublished). How low IGF-I levels might lead to increased risk of kidney cancer, as compared with other cancers thought to be positively associated (e.g., breast, colorectal, prostate), is not apparent from a biological perspective. IGF-I contributes to the regulation of glomerular filtration and kidney growth, and its administration has been shown to improve renal function and has been proposed as a possible therapeutic agent for patients with chronic renal failure (Hirschberg and Adler, 1998). Impedance in glomerular filtration rate and renal plasma flow may make the kidney more susceptible to carcinogenesis. IGF-I and IGFBP-3 are involved in an array of physiological and pathophysiological processes that could also impact risk.

Among the inherent strengths of our study is the ability to measure pre-diagnostic levels of IGFs, thereby supporting the temporal criterion for causality. Participants were followed for up to 12.7 years and all participants who were diagnosed with cancer within the first 5 years after the baseline blood collection were excluded, thus minimizing the possible influence of subclinical disease. Furthermore, as the kidneys are not a major source for production of IGFs, it is unlikely that the observed association reflects an effect of the disease. Because baseline information on factors known (or suspected) to modify the risk of kidney cancer existed in the parent study, we were able to elucidate the independent effects of IGF by multivariable adjustment.

A limitation of our study is that all participants were male smokers; therefore, the findings may not be generalizable to other populations, including women and nonsmokers, and the possible effects of smoking status (never, former, current smoker) on the observed associations cannot be examined. A potential concern is the higher proportion of men with hypertension among cases (28%). It may be that consistently elevated blood pressure promotes kidney cancer by causing damage to kidney tubules that may amplify the effect of IGF-I. Although we did not detect a significant interaction between hypertension and the IGF measures, and IGF concentrations were not significantly elevated among men reporting hypertension (after adjustment for age and BMI), the lack of statistical significance is likely contributed to by the small number of cases in this study.

In conclusion, our findings provide support for a relation between low IGF-I and kidney cancer risk, but require confirmation in larger, ethnically diverse populations that include women and nonsmokers.

## Figures and Tables

**Table 1:** Baseline characteristics (mean±s.d. or *n* (%)) by kidney cancer cases and comparison group

**Characteristic**	**Cases (*n*=100)**	**Comparison group (*n*=400)**	***P***
Age at randomization (years)	58±5	56±5	0.03^a^
Height (cm)	174.6±5.7	173.8±6.0	0.24
Weight (kg)	82.6±12.5	80.4±13.2	0.14
BMI (kg m^−2^)	27.1±3.7	26.6±3.9	0.28
Alcohol intake (g day^−1^)	7.5 (0–250)	11.4 (0–179)	0.05^b^
			
*Smoking history*			
Cigarettes per day	22.7±0.8	20.5±8.4	0.04^a^
Years smoked	35.6±0.7	34.8±8.5	0.04^a^
*Physical activity – leisure*			0.73
Light	43 (43.0)	172 (42.9)	—
Moderate	53 (53.0)	204 (51.1)	—
Heavy	4 (4.0)	24 (6.1)	—
			
*Medical history*			
Diabetes	8 (8.0)	28 (7.0)	0.73
Hypertension	28 (28.0)	74 (18.5)	0.04^c^
			
IGF-I (ng ml^−1^)	133 (34–291)	139 (45–348)	0.51
IGFBP-3 (ng ml^−1^)	2412 (952–4174)	2334 (714–4211)	0.60
IGF-I/IGFBP-3 molar ratio	0.21 (0.10–0.39)	0.22 (0.08–0.46)	0.11

Abbreviations: BMI=body mass index; IGFBP-3=insulin-like growth factor binding protein-3; IGF-I=insulin-like growth factor-I.

Medians (ranges) are reported for measures of insulin-like growth factors and alcohol intake.

Diabetes=history of diabetes and/or fasting glucose ⩾126 mg per 100 ml; hypertension=history of hypertension, elevated blood pressure.

a Significant based on Studentized *t*-test.

b Significant based on Wilcoxon rank sum.

c Significant based on χ^2^.

**Table 2:** Characteristics by quartiles of IGF-I and IGFBP-3 among comparison group

	**IGF-I**					**IGFBP-3**				
**Characteristic**	**Q1**	**Q2**	**Q3**	**Q4**	***P***	**Q1**	**Q2**	**Q3**	**Q4**	***P***
Age (years)	57.0	57.2	55.9	55.4	0.02	57.8	57.1	55.6	55.0	<0.01
Height (cm)	172.7	173.4	173.4	175.7	<0.01	172.2	174.0	173.8	175.3	<0.01
Weight (kg)	77.1	81.4	79.8	83.5	0.01	76.2	79.0	81.4	85.2	<0.01
BMI (kg m^−2^)	25.8	27.0	26.5	27.0	0.09	25.7	26.1	26.9	27.7	<0.01
Alcohol intake (g day^−1^)	19.9	12.2	9.3	12.2	0.42	10.3	14.6	10.0	12.2	0.96
Cigarettes per day	21.2	20.5	20.2	20.0	0.74	21.0	19.7	20.7	20.5	0.73
Years smoked	36.2	35.2	33.8	34.2	0.19	37.3	36.0	32.8	33.3	<0.01
Leisure physical activity (moderate+/light)	52.0	56.0	59.0	61.0	0.60	47.0	59.0	63.0	59.0	0.12
Diabetes (yes/no)	11.0	8.0	5.0	4.0	0.20	10.0	7.0	4.0	7.0	0.43
Hypertension (yes/no)	12.0	19.0	20.0	23.0	0.23	13.0	17.0	20.0	24.0	0.23
IGF-I (ng ml^−1^)	93.6	125.3	153.6	207.2	<0.01	106.6	129.0	155.3	188.4	<0.01
IGFBP-3 (ng ml^−1^)	1830	2178	2424	2972	<0.01	1694	2166	2546	3197	<0.01

Abbreviations: BMI=body mass index; IGFBP-3=insulin-like growth factor binding protein-3; IGF-I=insulin-like growth factor-I.

Medians are reported for measures of IGF and alcohol intake.

*P*-values based on analysis of variance, Kruskal–Wallis, and χ^2^-tests.
